# Méningite à *Streptococcus pneumoniae* sérotype 7A chez un nourrisson immunisé par deux doses du vaccin pneumococcique conjugué 13-valent: à propos d’un cas

**DOI:** 10.11604/pamj.2019.32.203.18157

**Published:** 2019-04-26

**Authors:** Kaoutar Ouazzani Touhami, Hind El Bayed Sakkali, Fakhreddine Maaloum, Idrissa Diawara, Makine Ouazzani Touhami, Mohamed Bezzari, Khalid Zerouali, Houria Belabbes

**Affiliations:** 1 Laboratoire de Bactériologie, Virologie et Hygiène Hospitalière, Centre Hospitalier Universitaire Ibn Rochd, Casablanca, Maroc; 2 Laboratoire de Bactériologie, Faculté de Médecine et de Pharmacie de Casablanca, Casablanca, Maroc; 3 Université des Sciences de la Santé Mohammed VI (UM6SS), Casablanca, Maroc; 4 Service de Pédiatrie, secteur libéral, Clinique Californie, Casablanca, Maroc; 5 Laboratoire Casalab, Casablanca, Maroc

**Keywords:** Streptococcus pneumoniae, sérotype 7A, méningite, ponction lombaire, vaccin pneumococcique conjugué 13-valent, Streptococcus pneumoniae serotype 7A, meningitis, lumbar puncture, 13-valent pneumococcal conjugate vaccine

## Abstract

Cause majeure de morbidité et mortalité, les méningites à pneumocoque (PNO) représentent un fléau mondial. Au Maroc, le vaccin conjugué 13-valent contre le pneumocoque (PCV13) a été introduit dans le Programme National de Vaccination en octobre 2010 selon le calendrier 2 + 1 et remplacé par le PCV10 en juillet 2012, selon le même calendrier. Malgré le recours au PCV13, essentiel dans la lutte contre les maladies pneumococciques, l'émergence de nouveaux sérotypes non vaccinaux entrainent toujours des méningites chez l'enfant et engendrent de lourdes séquelles. Nous rapportons le cas d'une méningite causée par *Streptococcus pneumoniae* sérotype 7A chez un nourrisson immunisé avec 2 doses de PCV13. La particularité de cette observation réside dans une méningite à PNO sérotype 7A, non contenu dans le PCV13, chez un nourrisson vacciné par 2 doses du PCV13. Les auteurs insistent sur la nécessité et l'importance d'un observatoire PNO et d'une large étude épidémiologique afin de déterminer les sérotypes en circulation au Maroc depuis l'introduction du PCV13 puis PCV10.

## Abstract (en)

Pneumococcal meningitis is a global scourge. It is a major cause of morbidity and mortality. In Morocco, 13-valent pneumococcal conjugate vaccine (PCV13) was introduced into the National Immunization Program in October 2010 according to the immunization schedule 2 + 1 and replaced by PCV10 in July 2012, according to the same schedule. Despite the use of the PCV13, which is essential in the fight against pneumococcal disease, the emergence of new non-vaccine serotypes always results in meningitis in children, causing serious sequelae. We report the case of an infant vaccinated with two doses of PCV13 with meningitis caused by Streptococcus pneumoniae serotype 7a. The peculiarity of this case study lies in pneumococcal meningitis due to Streptococcus pneumoniae serotype 7a not included in the PCV13 in an infant immunized by 2 doses of PCV13. We here insist on the need and the importance of an observatory for pneumococcal meningitis and of a wide epidemiological study in order to determine the serotypes in Morocco after the introduction of PCV13 and then of PCV10.

## Introduction

Les méningites à pneumocoque (PNO) représentent un fléau mondial pour les enfants de moins de 5 ans. Elles causent une morbidité et mortalité importantes [1]. En 2005, l'OMS a estimé à 1,6 millions de décès par an [2]. *Streptococcus pneumoniae* est une cause majeure d'infections communautaires invasives chez les nourrissons et les enfants. Les infections invasives à pneumocoque, notamment les méningites, restent graves, avec un taux de mortalité supérieur à 8% et un risque élevé de séquelles. Parmi plus de 90 sérotypes, seul un nombre limité compte pour les maladies pneumococciques. L'incidence du sérotype peut varier selon l'âge du patient, la région géographique et l'heure de la surveillance. L'avènement et la mise en place de vaccins anti-pneumococciques conjugués (PCV) ont permis d'accomplir des progrès considérables dans la prévention des méningites à PNO [3]. Au Maroc, le vaccin conjugué 13-valent contre le pneumocoque (PCV13) a été introduit dans le Programme National de Vaccination en octobre 2010 selon le calendrier 2 + 1 pour la prévention des infections pneumococciques causées par des sérotypes vaccinaux, tels que le 4, 6B, 9V, 14, 18C, 19F, 23F (inclus dans le PCV7) plus le 1, 3, 5, 6A, 7F et 19A. Le PCV13 a été remplacé par le PCV10 en juillet 2012, selon le même calendrier [4]. Avant l'introduction du PCV, les sérotypes inclus dans PCV10 et PCV13 couvraient respectivement 71,6 et 82,4% des sérotypes associés aux infections pneumococciques invasives chez les enfants de moins de cinq ans à Casablanca [5]. Malgré le recours au PCV, efficace dès les premiers mois de vie et essentiel dans la lutte contre les maladies pneumococciques, l'émergence de nouveaux sérotypes non vaccinaux entrainent toujours des méningites chez l'enfant et engendrent de lourdes séquelles en particulier chez les nourrissons. La méningite à pneumocoque, chez l'enfant ou l'adulte, reste mal documentée au Maroc. Nous rapportons le cas d'une méningite causée par *Streptococcus pneumoniae* sérotype 7A chez un nourrisson de 10 mois immunisé avec 2 doses de PCV 13 à 2 mois et demi et 5 mois de vie.

## Patient et observation

Nourrisson de sexe masculin, âgé de 10 mois, issu d'une grossesse suivie avec accouchement par césarienne et bonne adaptation à la vie extra-utérine a été admis en urgence dans une clinique médicale privée pour fièvre, cris incessants, vomissements, fontanelle antérieure bombée et suspicion de méningite. A l'admission, il était fébrile (38°C), conscient mais hypotonique, pâle, hyperalgique et hypotrophique (6,8kg). L'hémodynamique périphérique était stable (TA: 100/60, Pouls: 140/min) et la respiration, ample et superficielle. On a noté une légère dyspnée mais pas de cyanose (SaO2: 98%). L'abdomen était souple et aucun signe déficitaire n'a été retenu. Par ailleurs, les antécédents médicaux personnels étaient nombreux chez ce patient, notamment une détresse respiratoire néonatale sur dysostose maxillo-mandibulaire avec division palatine opérée à l'âge de 6 mois, un syndrome dysmorphique facial avec regard particulier, strabisme, base du nez élargi, visage triangulaire, un micropénis, une ectopie testiculaire droite et un testicule gauche minuscule. Par ailleurs, une IRM cérébrale a été effectué à 8 mois et a mis en évidence un élargissement des sillons, des citernes et du système ventriculaire avec diminution de la substance blanche. A noter également, un séjour récent pendant 15 jours dans un cabanon face à la mer. Ses vaccinations étaient à jour selon le calendrier vaccinal marocain: BCG fait à J10 de vie, 3 doses de Pentaxim^®^ et Rotateq^®^, 2 doses de PCV13 respectivement à 2 mois et demi et 5 mois de vie, 1 dose d'Engérix B^®^, la 2^ème^ dose reportée du fait de la maladie actuelle. Le bilan biologique a montré une augmentation de la protéine C-réactive (CRP) plasmatique (534mg/L), une anémie (Hémoglobine: 9,7g/L), une hyperleucocytose (17350/mm^3^) à polynucléaires neutrophiles (PNN: 88%) et une hypersidérémie (739 μmol/L). Cependant, les plaquettes étaient normales (356000/mm^3^), les bilans hépatique et rénal normaux ainsi que les échographies abdominales et cardiaques. La radiographie pulmonaire ne montre pas de foyer parenchymateux, ni de pleurésie. La ponction lombaire a révélé des anomalies du liquide céphalo-rachidien (LCR) avec un liquide trouble. La chimie du LCR retrouve une hypoglycorrachie sévère (0.01 g/L) et une hyperprotéinorrachie importante (3,95g/L). L'examen cytobactériologique du LCR était positif, révélant une augmentation du nombre de leucocytes (282 éléments/mm^3^) dont 95% de PNN. L'examen direct met en évidence de nombreux cocci à Gram positif en diplocoques capsulés et la culture isole de nombreuses colonies du pneumocoque. La recherche d'antigènes solubles revient positive à *Streptococcus pneumoniae*. Le Pneumocoque a été identifié selon les techniques standard de bactériologie. La souche bactérienne a été transmise au Laboratoire de Bactériologie, Virologie et Hygiène Hospitalière du Centre Hospitalier Universitaire d'Ibn Rochd, pour sérotypage. Il a été réalisé par la méthode d'agglutination en utilisant le coffret Pneumo-Test-Latex^®^ (Statens Serum Institut) et par la technique de gonflement de la capsule en utilisant des antisérums monovalents spécifiques des sérotypes (Statens Serum Institute antisera, Copenhague Danemark). Il a permis de monter qu'il s'agit d'un sérotype 7A, non contenu dans le PCV13. Le test de sensibilité aux antibiotiques a été réalisé selon les recommandations du CA-SFM-EUCAST. Erythromycine, Tétracycline, Chloramphénicol, Triméthoprime-Sulfaméthoxazole et Amoxicilline sont les antibiotiques testés par méthode de diffusion sur gélose Mueller Hinton additionnée de 5% de sang de mouton. La sensibilité du pneumocoque à la pénicilline G et la ceftriaxone est évaluée par mesure de la concentration minimale inhibitrice (CMI) par E-test. La réalisation de ce dernier à la pénicilline G et à la ceftriaxone a révélé une souche sensible avec un taux respectivement inférieur à 0,06mg/L et 0.5 mg/L. L'évolution de la méningite est favorable à J4 sous triantibiothérapie IV (amoxicilline+C3G+aminoside): l'examen direct et la culture du LCR reviendront négatifs. Par contre cette évolution va être emmaillée par la suite de plusieurs complications, notamment: 1) une gastro-entérite à Rotavirus d'évolution favorable malgré la vaccination par le Rotateq^®^ (3 doses reçues à 1 mois d'intervalle) ; 2) un syndrome d'hyperthermie majeure; 3) un état de mal convulsif; 4) une détresse respiratoire ayant nécessité une intubation trachéale et une ventilation assistée prolongée; 5) des signes biologiques d'infection bactérienne sévère (hyperleucocytose, thrombopénie, CRP et procalcitonine (PCT) élevées); 6)des désordres ioniques et biologiques importants (CIVD, anémie, hypoprotidémie); 7) une septicémie à *Escherichia coli, Klebsiella pneumoniae*, sécréteurs de béta-lactamase à spectre élargi (BLSE) et *Pseudomonas aeruginosa* multi résistants qui ont nécessité de multiples antibiotiques en fonction de la virulence et des différents antibiogrammes retenus notamment vancomycine, imipenème, colistine ou encore ciprofloxacine; 8) une atteinte pulmonaire sévère: atélectasie du poumon droit et foyer de condensation du lobe supérieur droit; 9) des signes d'encéphalite et collection sous durale liquidienne fronto pariétale périphérique gauche à l'IRM avec majoration de la dilatation ventriculaire lors du contrôle 15 jours plus tard. Le nourrisson sort après une longue hospitalisation de 32 jours. Si la méningite à PNO et les différentes septicémies ont guéri aux prix de multiples antibiotiques et si le nourrisson a pu être extubé, le pronostic reste péjoratif du fait du terrain précaire des complications de la méningite à PNO (ventriculite et dilatation ventriculaire) et de l'état respiratoire (atélectasie) nécessitant des soins et une surveillance continue.

## Discussion

*Streptococcus pneumoniae* est une bactérie capsulée qui contient plus de 90 sérotypes connus, tous différents les uns des autres en terme de prévalence et de virulence. Par conséquent, les sérotypes du PCV13 ne représentent qu'une partie mineure du pathogène. Auparavant, la vaccination par le PCV7 avait entrainé une réduction considérable des infections pneumococciques graves dans le monde entier [6-9]. Au Maroc, notamment à Casablanca, la plus forte diminution du taux d'incidence des infections pneumococciques s'est produite chez les enfants de moins de 2 ans, passant de 34,6 à 13.5 pour 100 000 habitants, respectivement avant et après vaccination [10]. En France, leur incidence a été réduite de 32% pour la méningite aiguë et de 38% pour la bactériémie [11]. Cependant, la même étude a également rapporté une augmentation considérable de l'incidence de la méningite et de la bactériémie due au sérotype 19A, non contenu dans le PCV7, chez les jeunes enfants [6,11]. L'essentiel des critiques formulées contre le PCV 7 était donc principalement lié à l'émergence de sérotypes non vaccinaux, bien que l'échec de la vaccination ait également été rapporté chez des enfants bien vaccinés ou partiellement vaccinés [12, 13]. Par contre, les études d'immunogénicité réalisées après l'introduction du PCV13 dans les calendriers de vaccination, ont montré que ce vaccin conjugué était capable d'induire une production d'anticorps, avec une bonne couverture vaccinale particulièrement contre six sérotypes (1,3,5,6A,7F et 19A): elle atteint 96,8% à 100% des enfants ayant reçu 3 injections [14]. Cependant, deux cas d'échec du PCV13 ont été rapportés par Godot *et al.* [9] chez un enfant âgé de 15 mois qui avait reçu 3 doses de PCV13 et n'avait aucune condition prédisposante sous-jacente liée à son échec vaccinal. Le deuxième cas, âgé de 2 ans et demi, avait reçu 3 doses de PCV7 et une dose de rappel de PCV13, cependant il était porteur d'un implant cochléaire. Notre patient, atteint d'une méningite à *S. pneumoniae* de sérotype 7A à l'âge de 10 mois, après avoir été vacciné avec deux doses de PCV13, avait cependant des conditions prédisposantes sous-jacentes: syndrome dysmorphique facial, anomalies des organes génitaux externes, dysostose maxillo-mandibulaire avec division palatine Il est bien connu que les infections sévères dues aux bactéries encapsulées sont courantes chez les enfants avec des conditions prédisposantes sous-jacentes connues telles que l'asplénie, une déficience du système du complément ou encore des déficiences en immunoglobulines. Notre patient a été examiné et après la réalisation de nombreux tests de laboratoire aucun de ces facteurs n'ont été trouvé. Bien que le PCV 13 contribue à réduire les infections pneumococciques sévères, la probabilité d'émergence de sérotypes non vaccinaux ne peut être exclue. En outre, les cas d'échec restent relativement rares en ce qui concerne l'étendue de la couverture réussie dans la grande majorité de la population infantile [3].

Au Maroc, peu de données sont disponibles sur le portage du pneumocoque et les sérotypes circulants. De plus, le PCV13 a été introduit dans le programme national de vaccination en octobre 2010 selon le calendrier 2 + 1, puis a été remplacé par le PCV10 en juillet 2012, selon le même calendrier [4]. Dans le secteur public, les nourrissons reçoivent le PCV10, alors que dans le secteur libéral, c'est le PCV13 qui est le plus utilisé [15]. Ainsi, cohabitent des enfants vaccinés par le PCV10 et d'autres vaccinés par le PCV13. Cependant, l'impact similaire des deux PCVs a fait l'objet de nombreuses reconnaissances internationales. En effet, l'Organisation pan-américaine de la santé (PAHO) déclare qu'aucun des deux vaccins (PCV10 ou PCV13) n'est supérieur à l'autre : les données de sécurité, d'efficacité sur le terrain et d'impact sont similaires [16]. La revue systématique latino-américaine des PCVs chez les enfants de moins de 5 ans affirme que l'impact global sur les incidences des maladies invasives à pneumocoque n'était pas statistiquement différent ou indépendamment du vaccin utilisé (PCV10 ou PCV13) [17]. Le centre international d'accès aux vaccins (IVAC) annonce que le PCV10 et PCV13 ont montré un impact comparable sur la maladie dans le cadre d'utilisation de routine [18] et enfin le groupe de conseil stratégique d'experts (SAGE) de l'Organisation Mondiale de la Santé (OMS) ont révélé qu'il n'y a actuellement aucune preuve de différence entre les deux vaccins sur l'impact global de la maladie [19]. De plus, l'étude FinIP qui est le plus grand essai randomisé jamais conduit avec les vaccins pneumococciques conjugués chez les enfants, a permis d'évaluer l'efficacité du PCV10 vis-à-vis des maladies pneumococciques invasives confirmées par culture dues à l'un des dix sérotypes vaccinaux pour les schémas de vaccination 3+1 et 2+1 chez des enfants ayant reçu au moins une dose avant l'âge de 7 mois [20]. Cette étude a montré que le vaccin était efficace dans 100%, 92% et 93% respectivement pour les schémas 3+1, 2+1 et les schémas 3+1 et 2+1 combinés [20]. Dans ce contexte, il apparait pertinent de mettre en place une large étude sur la surveillance des pneumocoques afin d'analyser les sérotypes circulants dans chaque population d'enfants vaccinés soit par le PCV10, soit par le PCV 13. Une étude, dans ce sens, est en cours de réalisation à Casablanca par l'Association Casablancaise des Pédiatres Privés (ACPP) en collaboration avec l'Association Clinique et Thérapeutique Infantile du Val de Marne (ACTIV) et la Société Marocaine d'Infectiologie Pédiatrique et de Vaccinologie (SOMIPEV).

## Conclusion

La particularité de cette observation réside dans une méningite à PNO sérotype 7A, non contenu dans le PCV 13, chez un nourrisson vacciné par 2 doses de PCV 13. Les auteurs insistent sur la nécessité et l'importance d'un observatoire PNO et d'une large étude épidémiologique afin de déterminer les sérotypes de PNO en circulation au Maroc depuis l'introduction du PCV 10 et PCV 13. Elle rappelle la nécessité pour les cliniciens de rester vigilants et de maintenir un haut niveau de suspicion chez les enfants, en particulier ceux qui présentent des conditions prédisposantes sous-jacentes mais également l'importance de la collaboration étroite entre cliniciens et biologistes.
